# 

**Published:** 1892-02

**Authors:** 


### CONTENTS:

ORIGINAL ARTICLES: PAGE

Notes on Parasites. By Charles W. Stiles, Ph. D. .... 65
Are Aloes Admissible in Colics. By H. R. Macauley, V. S. .... 67
Quittor or Quitter. By Charles Williams, V. M. D. .... 71
Corn Stalk Disease. By H. F. Spencer, D. V. S. .... 83
Paralysis. By Dr. J. M. Chase .... 85
The Inspection of Veal. By C. H. Magill, V. M. D. .... 89
Shoulder Lump. By Wm. Stinson, V. S. .... 90
Contribution to the Study of Mallein .... 91
Review of Recent Publications in Medical Zoology. By C. W. Stiles, Ph. D. .. 101
The Committee Report on Meat Inspection. Olof Schwarzkopff .... 107
Actinomycosis (Lumpy Jaw). By R. W. Hickman, V. M. D. .... 110

EDITORIAL:

Dignity of a Professional Journal .... 116
Change of Address .... 117
United States Veterinary Medical Association Comitia Minora .... 117
Recent Patents .... 118

NECROLOGY:

George Bridges .... 120

J. B. Killock .... 121

COLLEGES:

Ontario Veterinary College .... 122

SOCIETY PROCEEDINGS:

California State Veterinary Medical Association .... 123
The Connecticut Veterinary Medical Association .... 125
North Eastern Iowa Veterinary Medical Association .... 125
Ohio State Veterinary Medical Association .... 126
New York State Veterinary Medical Association .... 127
Society for the Study of Comparative Psycology, of McGill University, Montreal .... 135
Keystone Veterinary Medical Association .... 138
Massachusetts Veterinary Medical Association .... 139
Ontario Veterinary Medical Association .... 139
Philadelphia Society of Veterinary Medicine .... 140

REVIEWS: 141

